# Personal Identity Established by the Teeth; the Dentist a Scientific Expert

**Published:** 1884-01

**Authors:** Richard Grady

**Affiliations:** Baltimore, Md.


AMERICAN JOURNAL

OF

DENTAL SCIENCE.

VOL. XVII. THIRD SERIES—JANUARY, 1884. No. 9.

ARTICLE I.

### PERSONAL IDENTITY ESTABLISHED BY THE TEETH: THE DENTIST A SCIENTIFIC EXPERT.

BY RICHARD GRADY, D. D. S., OF BALTIMORE, MD.

Few words are needed to introduce this article. In the present advanced stage of dental science, the entire absence of this topic from dental text-books must be noticed with regret. Their authors should constantly endeavor to bear in mind a truth which those engaged in education sometimes forget, that what is well known to them may be new to others. Forensic Medicine embraced in a general medical education has been considered scarcely needful to the dentist. In fact, the dentist is puzzled to know whether he is practicing a specialty of the great domain of medicine, or a distinct science and art independently standing on its own merits. Nothing having heretofore been done, so far as known, to group together cases in which the teeth have been used as evidence in disputed identity and those in which dentists have given scientific testimony, this paper is prepared as a pioneering effort, made under difficulties. It is hoped that it may prove an acceptable addition to

Read in substance before the Maryland and District of Columbia Dental Association, at the meeting in Baltimore, October, 1883.

Dental Literature, and give the reader a desire to extend his inquiries into the same interesting field.

DENTAL JURISPRUDENCE should have not only "a name but a local habitation." Now that it has been dignified and fostered elsewhere, we want it to be recognized in our Colleges and Universities, which assuredly ought to be the case. It is a subject of present and prospective importance, and becomes a desideratum, almost a necessity, to every intelligent dentist. In the cases cited, principles have been suggested and confirmed, and methods have been determined by which future investigations in this branch of knowledge may be successfully prosecuted. The learning, the sound judgment, the self-explaining order and minutely-traced ramifications which characterize the testimony of those quoted will, it is believed, be acknowledged as honorable to the profession. Their truths, indisputable and demonstrable, though greatly involved, gave the scientific world a more correct idea of the dentist. In no instance did future experience prove the falsity or unreliability of their conclusions. Dr. Nathan C. Keep's careful and explicit testimony in the Webster-Parkman case furnished the strongest evidence of the identity of Dr. Parkman that was brought out; and, in the Goss case, the exact learning and nice discrimination of the professional writer and scholar, Dr. Ferdinand J. S. Gorgas, in the preparation of the detailed statement of the examination of the maxillary bones and teeth of the body exhumed, resulted in the insurance companies being advised that it would be impossible to reconcile the dissimilarity between the diseased jaws and mouth of the almost toothless corpse, and the mouth of W. S. Goss as described by his wife.

Identification is not so easy as we are apt to believe. If asked to point out the feature or features by which your most intimate friend is distinguished, you probably could not do it. Were you to refer to the size of his head, shape of his face, nose or mouth, you would doubtless find that in all these respects, he is not singular. It must be admitted then, that the subject of personal identity is one particularly beset with difficulties and perplexities. Undoubtedly, there is a large number of persons, it may be even a majority, whose evidence upon any question of identity though perfectly honest is untrustworthy. Such persons are untrained in minute observation. When, therefore, there is a question as to any point of science or art the opinion upon that point of persons specially skilled in such matters is sought, division of labor making men expert in their several avocations.

In several cases of deep and startling interest, found in Forensic Literature, identity has been proven in regard to persons or things by the dentist as a scientific witness. This proof required thought and acumen, powers of observation, knowledge of facts, habits of induction. The attention of the profession should therefore be called to the importance dentistry assumed in these cases and to the probable necessity of being prepared for similar ones. It may be well to add that the dentist when called to give expert testimony cannot, on the strict principles of law, be cross-examined as to the opinions of others, for that would be hearsay. He is called to give his own opinion, and not to say who agrees or who disagrees with him. He is thus a representative man. He has not merely a personal interest in acquitting himself creditably and honorably, his individual interests do not alone suffer. But every blunder he commits and every unprofessional or undignified act he performs reflects with damaging force on the profession as a whole. On the contrary, if he brings thought, learning, judgment, discrimination, common-sense to bear on the case, dentistry is honored, and commands respect among kindred professions. And as events of the greatest moment are very often brought about by causes apparently the most trivial, he must never forget that imprisonment or freedom, life or death may be involved in his evidence ; the ruin of a fellow being and a harvest of self-reproach.

Questions of identity may turn (as will be shown) on the absence or presence of teeth, or on the condition of the alveolar process as indicative of the period when the teeth had been removed, or on the presence of artificial teeth and mechanical appliances for retaining them, or on certain dental peculiarities—arrangement of teeth, state of decay, worn appearance. A cast of the mouth may be of great service in evidence and set at rest questions respecting the teeth and the jaws. The perfection of dentistry in making records by moulds was exhibited in an interesting manner in the Webster-Parkman case, and rendered plain to judge and jury what simple description might have failed to do. The silent evidence of a mineral tooth sliding into the matrix in which it was made, and fitting no other place, amounted to demonstration. Plaster casts properly labelled should be preserved and peculiarities of all kinds noted. A doubtful case tried at Edinburgh was decided by a dentist who produced a cast of the gums which he had taken before death. The graphic form of evidence by sketch illustrations also commends itself to all interested in a case. Prof. R. B. Winder, of Baltimore, has throughout his practice so carefully studied his patients that, guided by a record he has kept, he claims that he could make illustrative drawings of their jaws and teeth in case of doubt or discussion. Cremation, as suggested by certain enthusiasts, will, however, put an undoubted barrier to a dead body's furnishing evidence to assist justice (as earth burial so often permits) either in clearing the innocent or punishing the guilty.

Before we proceed to the cases without which this paper would be incomplete, we must permit ourselves a cursory glance at the following:

*1. Indian Medical Gazette, 1875.* Identity determined from a skull, five ribs and five vertebræ. The teeth and the peculiar shape of the skull were the important data in this case.

*2. Taylor's Medical Jurisprudence, Vol. 1.* A certain woman (named Caroline Walsh) disappeared. It was believed that she had been "burked" for an anatomical school. A woman named Ross was tried for the murder.

On the same day that Walsh disappeared, a woman (named Caroline *Welsh*) was found on the street with her hip broken. The contention of the defence was *Welsh* was the woman Walsh that the prisoner was accused of murdering. It was proven that

Walsh was from Kilkenny was eighty-four years of age, tall, of sallow complexion, having gray hair, and *very perfect incisor teeth.*

*Welsh* came from Waterford, was sixty years of age, tall, of dark complexion and had *no front teeth, the alveolar cavities probably having been absorbed for some time.*

*3. Wood's Legal Medicine, Vol. 1.* Body exhumed after eleven years. The length of the hair and the state of the teeth satisfactorily established identity also.

*4. Case at Versailles.* Body exhumed after three years. Identity proven by the disposition of the teeth.

*5. Sargent's History of Braddock's Expedition.* Identification by means of an artificial tooth. Sir Peter Halket in 1758, after the reduction of Fort Du Quesne, proceeded to the spot of Braddock's defeat to discover, if possible, the remains of his father who was there killed. In reply to his anxious questions one of his tawny guides had already told Halket that he recollected, during the combat to have seen an officer fall beneath such a remarkable tree as he should have no difficulty in recognizing; and at the same moment another, rushing to his side, was instantly shot down and fell across his comrade's body. As they drew near the spot, the detachment was halted, and the Indians peered about through the trees to recall their memories of the scene. With speaking gesture, they briefly discoursed in their own tongue. Suddenly, and with a shrill cry, the Indian of whom we have spoken sprang to the well-remembered tree. In a moment, two gaunt skeletons were exposed lying together, the one upon the other, as they had died. No sign remained to distinguish the relics from the hundred others that strewed the ground. At the moment, Sir Peter remembered him of a peculiar artificial tooth which his father bore. The bones were then separated, and an examination of those which lay undermost at once solved all donbts—"It is my father!" exclaimed the unhappy youth, as he sunk into the arms of his scarcely less affected friends.

6. *Taylor's Medical Jurisprudence.* Supposed murder of Lydia Atlee. In this case, a body was dug up after fourteen years interment. It was believed to be that of Lydia Atlee. It was proved that the deceased had a tooth (first molar on left side) removed a fortnight before death, and this was found wanting in the jaw recovered. The cavity was partly filled up which might be explained by the tooth having been extracted without the removal of its fangs.

7. *Guy's Principles of Forensic Medicine.* The remains of the body of the Marchioness of Salisbury discovered among the ruins of the Hatfield House were identified by the bone having gold appendages for artificial teeth.

8. The reports of cases on the continent of Europe are deeply interesting on account of the method of investigation· A priest in Germany was charged with murder. Eight years after the murder an examination was ordered. (He had already undergone *eighty* examinations.) It began at four in the afternoon and was prolonged to *midnight.* The judge addressed himself to the conscience of the prisoner, and, after concluding an impassioned appeal, he suddenly raised a cloth from the table under which lay a skull placed upon a black cushion. "This," said he, "is the skull of———, which you may still recognize by the two rows of white teeth in the jaws. (The deceased had been remarkable for the beauty of her teeth.) The priest rose instantly from his chair, stared wide upon the judge, retired a step or two, so as to hide the object from his eyes, and pointing to the skull replied, "My conscience is calm. If that skull could speak, it would say, 'The priest is not my murderer." Suffice it to say that two years after, he confessed, quoting the doctrine which holds it to be "lawfui to take away the life of another when there exists no other way of preserving our reputation ; for reputation is dearer than life itself."

9. In August 1817, a master-miller disappeared in Germany. Not until October of the same year did his wife inform the provincial magistrate. In about a year it was rumored abroad that he had been murdered in the mill. The body had been buried among some rocks. Under a layer of leaves and moss were found part of a skull, several ribs and other bones, pronounced to be those of a man. The man who helped bury the body said "These must be the bones of the murdered ——, for his sons brought his body here in my presence four years ago, and threw it into this cleft. We then covered it with leaves and moss. Moreover —had remarkably fine teeth, just like those in the jaw bone before us."

10. In 1827, a young woman named Maria Marten left her father's house to be married to a William Corder. Time passed, and the Marten family heard nothing of them. Anxiety led to suspicion, and probably led the stepmother to dream, as it is reported that she did on several occasions, that Maria lay buried in a certain barn. Eleven months after she left her home, the barn was searched. Upon lifting up the straw there were appearances of the earth's having been disturbed, and a further search resulted in the discovery of a dead body, which in spite of the lapse of time was recognized by the want of certain teeth as that of the unfortunate Maria Marten.

"But to pass from these on which, after all, we have only touched, we would now in order fully to show the value of our own distinctive jurisprudence refer to the Webster-Parkman case in Boston, and the Goss-Udderzook tragedy in Baltimore. They commend themselves to our special notice because dentists assisted in them.

11. The trial of Professor Webster is one of the most remarkable cases in the annals of criminal jurisprudence. On the 24th of November, 1849, Dr. George Parkman, a well known benefactor of Boston disappeared. Search was made for him by his friends and the police. As he was the owner of many tenements, the rent of which he usually collected himself, it was feared that some of the rough tenants had robbed him, and then murdered him. The most active and energetic efforts were induced by large rewards and by the general interest which was felt in the mysterious disappearance. Nothing was learned except that he was seen at the Medical College of Harvard University, in the company of Dr. John W. Webster, Professor of Chemistry there, on the afternoon of his disappearance.

A week after this, search having been made in other places, the body was looked for where the living man was last known to have been, an impression or a prejudice having arisen that the walls of the College held the secret. The officers who conducted the search were surprised to find in a vault in Dr. Webster's laboratory, parts of a human body, and with them some towels marked with the initials of Dr. Webster. Further search led to the finding in a tea chest almost the entire trunk of a human body. There were also found in a furnace a great number of pieces of bones belonging to a human body, which were mutilated. These remains were submitted to the examination of medical and scientific men. The parts found were put together. There were missing the head, the arms, both feet and the right leg below the knee. Not one fragment was met with that could be called a duplicate—that is, the whole when placed together, showed that they belonged to different parts of one human body, and could not belong to two or more bodies. There was nothing in this fact however to identify the remains as those of Dr. Parkman. They were in a Medical College where the dissection of dead bodies was regularly practiced in the study of anatomy and kindred branches.

But among the ashes of the furnace were about 174 grains of gold, valued at $6.94, a pearl button partially calcined, a human tooth that had a cavity in it as if once filled by a dental operation, three blocks of mineral teeth with rivets but without the gold plate in which they are set; a great many fragments of bone belonging to the skull (four fragments of the lower jaw were specially noticed). These bones appeared to have been exposed to intense heat and some of them in contact with gold being colored pink by the oxide of that metal.

Dr. Keep, the family dentist of Dr. Parkman for twenty-five years, examined the mineral teeth, and pronounced them his work—the same which he had made three years before for Dr. Parkman. The parts of the jaw found in the furnace were put together and were found to fit a mould of Dr. Parkman's jaw which had been taken by Dr. Keep when making the artificial teeth. The jaw was very peculiar in form and the coincidence of these bones with the mould was too remarkable to admit of much question whether or not it was the jaw of Dr. Parkman. The form of the lower jaw indicated by four fragments of the right half implied a rising chin, which was so pronounced a feature of Dr. Parkman, that one of the witnesses stated that in jest with her sister she had called him "chin." There was also a remarkable "depression" on the left side of the lower jaw, corresponding with what the family dentist called "a great irregularity." It was claimed that if Dr. Keep saw these same mineral teeth anywhere, even beyond the seas, he would be able to prove that he had made them for Dr. Parkman, such were their peculiar characteristics; and further, that the bone of Dr. Parkman's jaw had a peculiarity about it that no other human being could have.

How little could it have been thought when three years before Dr. Parkman was hailed by a crowd of spectators as one of the founders of the medical college then first opened that that building was to be otherwise associated with his name becoming at once altar, tomb and funeral pile; and that the teeth which were to have helped his speech on the occasion of the festival should be only the silent, incorruptible witnesses of the murder!

The scientific report of four physicians appointed to examine the remains found in the Medical College, stated that the remains were those of a man of Dr. Parkman's height and general appearance, and about his age; the testimony of the demonstrator of anatomy showed that the remains were not parts of any subject used in the college for dissection. Dr. Parkman's agent testified "His jaw was prominent—the under part at least. I should not want to have it understood that I swear positively to the identity of the remains with the body of Dr. Parkman," and Dr. Parkman's brother-in-law testified; "I believe the remains to be the body of Dr. Parkman from the fact that the doctor was missing, as much as from the hairiness of the back. If he had not been missing I should not have thought anything about the peculiarity of the hair on the back."

But the testimony of Dr. Keep the dentist, and of his assistant, Dr. Lewis Noble, was the most striking part of the evidence by which the identification of the remains of the murdered Dr. Parkman was secured, and, although circumstantial evidence it was full and irresistible. We will now give an abstract of the evidence of these and the other dentists.

Dr. N. C. Keep, dentist, testified as follows :—

"Have been in the practice of dentistry for thirty years. Give attention both to natural and artificial teeth. Knew Dr. George Parkman as early as 1822. As early I think, as 1825, he employed me as his family dentist; and since that time whenever he needed assistance, I have been the person on whom he called. Was shown the block of mineral teeth by Dr. Lewis. I recognized them as the teeth I had made for Dr. Parkman in 1846. Dr. Parkman's mouth was a very peculiar mouth in many respects, differing in the relation that existed between the upper and lower jaw so peculiarly that the impression left upon my mind was very distinct. I remember the peculiarity of the lower jaw with great exactness.

"The circumstances connected with the teeth being ordered were somewhat peculiar. The first question asked by Dr. Parkman, when the teeth were ordered, was, 'How long will it take to make them?' I took the liberty to ask why he was so particular to know. He told me that the Medical Co ege was to be opened, and that it was necessary for him to be there, and perhaps to speak, and he wanted them by that time, or else he did not want them at all. That time was a very short one; the peculiarity of the mouth made it a case requiring as much skill as could be used. I began to do it as soon as possible; gave a large part of my attention to it from day to day. In consequence of these circumstances, and the shortness of the time, and the close application I gave to it, I remember very distinctly what was done, more than in ordinary cases. I proceeded in my usual mode, to take the impression. The first step was to take an exact *fac simile* of each jaw, with wax, metal, liquid plaster, &c. A plate was made from that, and the next step was, of course, to ascertain the relation between the upper and the lower jaw. A model of the lower jaw was made from an impression taken with wax, while in a plastic state and by means of this the lower plate was fitted. The upper plate was fitted in the same manner. (Dr. Keep exhibited the original plates, which fitted to the models.) These plates were made before the gold plates, to ascertain if there were any defect in the models. When the plates were fitted to his mouth, I requested him to close it until I satisfied myself as to the suitable distance.

"A great irregularity on the left side of the lower jaw of Dr. Parkman gave me great trouble in getting this up. Each set of teeth was made in three blocks, and then joined to the gold plate. There were spiral springs that connected the two sets of teeth, to enable the patient to open his mouth and close it with less danger of the teeth being displaced, than they would have been without the springs. There was an accident which injured one of the teeth in the front block, and delayed the finishing of them until near the end of the night before the opening of the Medical College. They were finally finished by setting my assistantat work on them with all the assiduity he could, at just thirty minutes before the opening of the Medical College. My assistant was Dr. Noble. When I next saw Dr. Parkman, he said that he did not feel that he had room for his tongue. In order to obviate that difficulty, I ground the block of the lower jaw on the inside, to make it lighter, and furnish more room for the tongue. This grinding, at that time, was not accomplished with so much ease. The teeth being on the plate, we could not grind on a large wheel. We had to grind on a very small wheel. This grinding removed the pink color that represented the gums, and also the enamel from the inside of the lower teeth. The shape left by the grinding was very peculiar, because of its being ground on a small wheel, smaller than itself.

"I saw Dr. Parkman frequently. The last time I saw him was, as near as I can remember, about two weeks previous to his disappearance. He called late in the evening, about ten o'clock He told me his trouble. I took his teeth, both upper and lower, examined them, and put on a new spring. I had no more professional intercourse with him at all. I was told that Dr. Lewis wanted to see me, and he presented me with these remains of mineral teeth (showing them), with the request that I should examine them. On looking at them, I recognized them to be the same teeth I had made for Dr. Parkman. The most of the upper portion that remained was the block belonging to the left side of the lower jaw. Several other parts had been very much injured by fire. I proceeded to look for the mould upon which these teeth were made, put the metal upon its proper place, and it fitted exactly. There is sufficient left of these blocks to identify the place where they belonged. There is no mistake. (He then showed the mould, and remains of teeth, &c) All the pieces having been found, there were five pieces, which fitted to their exact places. The only piece that could not be identified might or might not have been right; but it was supposed to be right, as there was no reason that it should not be so."

The blocks of teeth, &c., were here shown to the jury by the witness, and afterwards to the judges.

"I found imbedded, more or less, with these mineral teeth, some very minute portions of gold. I saw the teeth in the Doctor's head, the last time I saw him, in conversing with him. The presumption is very strong that these teeth were put in the fire in the head. Such is the nature of these mineral teeth, that, especially if they have been worn, they absorb small particles of water; when suddenly heated, the surface becomes closed and the water becomes steam, and there would be a report, with an explosion. I have known such explosions to take place, on heating teeth that have been worn; and when they have been worn recently, the explosion is always sure to take place, if heated rapidly. If, while in the head, they were put into the fire, only a small portion would be exposed to the heat; and as the temperature would be raised so gradually, the water would have time to escape; and this accounts, in my mind, for the teeth not being cracked, excepting the front teeth, which would have been most exposed. I have found, fused into the remains of teeth, portions of the natural jaw. All these teeth were exhibited to me at the same time."

Cross-examination. "My first impression, on seeing the teeth shown me by Dr. Lewis was of the circumstances which I have related. Do not think I have been burnishing up my recollection since they were shown me. Knew them myself, without examining the mould; but I did examine them with the mould. The mould of Dr. Parkman was preserved, as moulds usually are, for future use, in case of accident to the teeth.

Dr. Lester Noble, Dr. Keep's assistant testified: "I remember making mineral teeth for Dr. Parkman in 1846; wrote Dr. Parkman's name on the model, "Dr. Parkman, October, 1846." I recognized the teeth the moment I saw them, as well from the general configuration as from several peculiarities which I remembered; noticed also the defacement given them by Dr. Keep in grinding down the edges. Am positive these are the teeth made for Dr. Parkman; have as good reason to believe these teeth were made by me, as I have to believe any fact I know Remember that they were to have been done by the day that the Medical College was opened; remember the circumstances of the opening; I was present and watched to see if Dr. Parkman would speak, in order to discover how the teeth would work; he did not speak as I inferred he would when he was complimented by Governor Everett for his generosities.. I understood that Dr. Parkman had given the land on which the Medical College stood to Harvard University."

Another dentist (Dr. Daniel Harwood, practicing in Boston from 1839), stated that he was quite in the habit of distinguishing the works of one of his profession from those of another. He even went so far as to say "There are characteristics about teeth by which a dentist would be as likely to know his own works as a sculptor would be to recognize his own statue, or a merchant his own handwriting."

Another witness (Dr. Joshua Tucker, dentist in Boston for twenty-one years) said: "I think the dentist who made 'it' could identify it as easily as an artist who had spent a week in painting a man's face on canvas would recognize the picture painted by himself.

Taking all these together; finding the body hypothetically constructed by means of the science of anatomy from the discovered remains to correspond perfectly with that of Dr. Parkman in height and age, and the general form differing in no known respect; and finding further a very marked peculiarity in the bone of the lower jaw common to both, there seemed a great probability of identity which is raised to a sufficient certainty by Dr. Keep's unqualified recognition of the teeth.

The case triumphantly refutes the common objections as to the sufficiency of circumstantial evidence. The jury on the eleventh day of the trial agreed upon their verdict of guilty, and no one can pretend to doubt that it was just, for Dr. Webster finally, clearly, and unequivocally confessed it to be so. The confession verified in a remarkable degree the particulars of evidence given, showing how truly by a patient examination of facts and circumstances the history of a man's most secret hours may be read.

12. *Goss-Udderzook Tragedy.* This is a double story of fraud in the earlier stage and murder in the later, unique in outline and full of ingenuity in details. We have thought this case of sufficient importance to present a tolerably full abstract, since strangely it has heretofore been overlooked in Dental Literature.

February 3rd, 1872, a Baltimore newspaper stated that W. S. Goss, residing 314 N. Eutaw Street, had been burned to death in a cottage the previous night. This house was several miles out of the city, and the fire was supposed to have been caused by an explosion of chemicals, with which

Goss was experimenting in making a substitute for india-rubber. The house was entirely consumed. The remains of a human body were drawn out of the building, the lower limbs destroyed, and the features so burned or charred as to be beyond recognition. From the shape of the chest, neck and head, the corpse was identified as that of W. S. Goss. Indeed who else could any one suspect it to be? So the coroner held an inquest which rendered the verdict "That W. S. Goss came to his death by the explosion of an oil lamp."

The body was taken to Baltimore, and after solemn funeral services, removed to Baltimore cemetery. While it lay at Eutaw Street, the widow had no question as to the fact that the remains were those of her husband. She knew the contour of the neck, head and breast. Some ten or more witnesses testified to their belief in the same identity, regarding the recognition of the body as not difficult or doubtful.

In May, 1871, W. S. Goss seemed to be seized with a sudden mania for insuring his life. He had insurance to the amount of $25000 payable to his wife. His last policy was dated eight days prior to his "cremation." The stories of William E. Udderzook and A. C. Goss, brother-in-law and brother of W. S. Goss conveyed the impression that "they knew too much" and led the insurance companies at once to inquire into the facts. While all disclosures tended to strengthen the suspicion of fraud, there was absolutely nothing in the way of direct demonstration. The companies refusing to pay the claims at maturity, suits were promptly instituted under each policy.

At the inquest, it was observed that, although the extremities were more or less consumed, the head was entire, and it was believed the bones of the skull, including the teeth, were uninjured. Any peculiarity of the teeth whether natural or arising from mechanical dentistry, might at once determine the question of identity of these remains. An effort was made to obtain a description of any such peculiatriy if it existed, for the purpose indicated. In pursuance of this information every dentist in Baltimore was interrogated, but with only negative results. So far as could be ascertained, Goss was known to have unusually good teeth, which were conspicuous in his ordinary conversation, and were fully exposed when he laughed. From no source could it be learned that he had occasion to employ a dentist.

Mrs. Goss had testified before the coroner to certain facts touching the *personnel* of the supposed deceased She was therefore requested to make a more elaborate description, especially of his teeth, and to grant permission for the exhumation and examination of the remains. This was the proposition in regard to the teeth: Furnish "description of his teeth, their quality and appearance; whether wholly or partially sound or defective, natural or artificial; whether he had any peculiar teeth, had lost any, and how many and what teeth; had any teeth broken and how many and what teeth, and how broken, had any teeth filled or otherwise operated upon by a dentist? and how, where and when operated upon, and by what dentist." Her reply was: "He wore no artificial teeth to my knowledge, never complained of pain or inconvenience from decayed teeth, and I do not remember his requiring the services of a dentist during the time we lived together. I should call his front teeth quite regular."

The remains were exhumed and examined in the Baltimore College of Dental Surgery by Professor F. T. Miles, M. D.; R. Wysong, M. D.; Prof. E. Lloyd Howard, M. D.; and Prof. F. J. S. Gorgas, M. D., D. D. S. The medical experts who examined the exhumed body were able to say little that could throw light on its identity with W. S. Goss except in the matter of teeth. The physicians stated (1) The remains were those of a male. (2). He was not a negro. (3.) He was between the ages of twenty-five and fifty years. (4.) He was of fair average height, of stout build, and of great muscular strength. (5.) It is impossible to determine whether the burning was the cause of death or was post-mortem."

But Prof. Gorgas, the dentist, was able to give more valuable opinions—The maxillary bones and teeth were thus fully described by him.

CONDITION OF MAXILLARY OR JAW BONES.—*Superior Maxillary.*—Perfect, except margin of alveolar process. *Inferior Maxillary.*—A portion of the external surface of body of the bone below the alveolar process and to the right of the median line, including the right mental foramen, destroyed for a space of two and a half inches long, and one inch broad or wide; the bone otherwise perfect.

Number of teeth remaining in upper jaw, 2; number of teeth remaining in lower jaw (including one root of tooth), 7.

CONDITION OF THE TWO TEETH IN UPPER JAW.—*Superior Right Second Bicuspid.*—A superficial carious cavity on posterior proximal surface. Cusps on grinding surface worn away by mechanical abrasion, but not so much as wholly to obliterate the natural depressions on this surface. *Superior Right Third Molar.*—Perfectly sound.

CONDITION OF THE SEVEN TEETH IN LOWER JAW.—*Root of Inferior Right Central Incisor*—The crown evidently destroyed by caries to a point below free margin of the gum, before death. *Inferior Right Lateral Incisor*—Perfectly sound. *Inferior Right Canine*—Sound; angle worn away by mechanical abrasion. *Inferior Left Central Incisor*—Various cavities on both proximal surfaces, which communicated. *Inferior Left Canine.*—Carious cavity on the anterior proximal surface. *Inferior Left Second Bicuspid*—Small carious cavity on the anterior proximal surface. *Inferior Left Third Molar*—Large carious cavity on the buccal surface, near neck; also a superficial cavity on grinding surface, Grinding surface worn by mechanical abrasion, so as almost to obliterate the natural depressions on the surface.

*Form of Irregularity of Inferior Front Teeth*—Approximal surfaces of the inferior right lateral incisor and inferior left central incisor approach near together at the cutting edges; caused by the loss of the crown of the right central incisor, the root of this latter tooth remaining in the alveolar cavity.

No tokens of the wearing of any artificial teeth were discovered. This careful and critical examination could not be reconciled with the statement of Mrs. Goss describing the mouth of her husband. As Mrs. Goss had been married to W. S. Goss fourteen years, during which time they had lived together it was fair to presume she necessarily would have heard complaints of pain and inconvenience from such badly decayed teeth and jaws; that she would have remembered the required services of the dentist who had extracted so many of the teeth, and that she would not have called such front teeth "quite regular."

The trial began May 27th, 1873, in the Circuit Court of the United States. The defense was conducted by counsel of the several insurance companies interested—all of whom were members of the Baltimore bar. The descriptions of W. S. Goss given at the trial were so singularly consonant with one another as to show him to have been a man of very marked and noticeable form of face. Especially he was said to have had unusually large fine teeth. Upon the trial, then, these teeth formed the defendants' *piece de resistance* —Their witnesses stated that they had often noticed them when Goss talked or smiled.

The companies broke down in the attempt to prove that W. S. Goss had been alive since Feb. 2, 1872. Their counsel however came up manfully to the more difficult task of proving affirmatively that the corpse was that of some other person than the insured.

Prof. E. Lloyd Howard testified (in regard to body exhumed.) " Of the sixteen teeth belonging to the upper jaw, nine had been lost before death; by that I mean some time before death. There remained in the upper jaw two teeth; there had fallen out since death three teeth; and two sockets, which had once contained teeth were shallow, so that it was uncertain whether these teeth had been lost before or after death. Nine of the sixteen teeth were certainly lost long before death, and two others possibly were. One of the teeth lost in the upper jaw was a front tooth. Of the teeth belonging to the lower jaw, seven were lost long before death. One tooth had been partially destroyed by disease. One root of a tooth and eight teeth remained in the jaw. Of the seven teeth lost, six were back teeth, and one was a front tooth, and the one of which the root only remained was a front tooth. This would have given the appearance of two front teeth lost from the lower jaw.

Of the thirty-two teeth, sixteen were unquestionably lost before death, and of the sixteen remaining, one was only a root in the socket. The crowns of two of the front teeth approached each other, over where a tooth had been lost. In the upper jaw the palatine canal, which perforates the roof of the mouth just behind the two middle front teeth was greatly enlarged by an abscess, which had existed previous to death and which abscess communicated with the diseased cavity of one of the front teeth. The abscess appeared to have formed about the root of the tooth. In our opinion this abscess communicating with the cavity in the bone had absorbed or eaten through the bone to that extent forming an opening between the socket of the tooth and this anterior palatine canal. It must have been considerably diseased to have left such lesions in the bone. It could not have been otherwise than very painful. We judged from the facts pointed out that the other teeth over the diseased root must have approached each other, giving a crooked, irregular appearance. (Plaster model of mouth handed witness.) I have examined the model before and found it corresponded very accurately with the jaws we examined."

Prof. F. J. S. Gorgas, who united in the report of the examination of the remains, and had prepared plaster casts of the mouth, which were used by witnesses in their testimony relative to the teeth, was unable to testify, being absent on account of the illness of a daughter who was on a visit to a western city. Dr. Gorgas' evidence being unattainable, Dr. Robert Arthur was called for the defendants, and testified:

"I have practiced the profession of dentistry for thirty-two years. (Plaster models of the mouth of subject handed to witness.) The operations of nature, after a loss of teeth during life are such as to leave it a matter of no possible scientific doubt whether teeth have been lost before or after death, provided they have been lost a certain time before death. Looking at this model of the lower jaw, speaking as a scientific expert, I would say these teeth were lost, with the exception of the two ones from these two cavities (referring to the two where the teeth had fallen out since death,) certainly more than two years before the death of the subject. In this model of the upper jaw, three of the teeth, I should say, were recently lost. The tooth next to the front tooth has been lost unquestionably from one to two years. The absorption seems to have been complete, but the eye tooth and next to it seem not to have been lost so long; the absorption has not been completed. I should infer, from the small cavities that the front tooth had been lost some time before death. Obviously there was a great deal of disease here; there must have been much physical pain. This place where the penetration appears to have taken place in the roof of the mouth shows a perforation through the bone communicating with the socket of the teeth. The teeth must have been very much diseased to have got into this condition. Not within my experience have so many teeth been lost without the patient suffering great pain, and of necessity requiring the services of a dentist. In masticating ordinary food, the person must have found great difficulty. He must have eaten with great discomfort. I would not by any means call the person's front teeth "quite regular." Teeth that are absent could scarcely be called regular. Even the teeth of the lower jaw must have presented a very irregular appearance."

Dr. Charles H. Ohr, of Cumberland. (Plaster casts of the mouth of the exhumed subject handed to witness.) "It was a very irregular set. In my judgment he required the services of a dentist on more occasions than one, and had suffered a great deal of pain on account of diseased teeth There is very little surface here for mastication, or chewing of food. The grinding teeth are not opposite each other in such a way as to enable the person to masticate ordinary usual food. The abscess at the roof of the mouth would have produced intense pain.

The case was given to the Jury who returned a verdict in favor of Mrs. Goss, for full amount of insurance with interest added. Defendants counsel gave notice of motion for a new trial.

This was the end of the first act. Tragic events followed. The verdict was rendered June 6th, 1873. Almost directly after this (July 1st or 2d,) the motion still pending, news came that the body of a murdered man had been discovered in Chester County, Pennsylvania. The story was that William E. Udderzook arrived there June 30th, accompanied by a man whom Udderzook spoke of as his friend, but did not mention any name. A jury of inquest found "That the same man (name unknown) came to his death from wounds inflicted by a dirk knife or other sharp instrument in the hands of William E. Udderzook, of Baltimore, Maryland.

The fact of Udderzook having been principal witness of the fire on the York road, coupled with the fact that the remains of the missing stranger bore a striking resemblance to the description of Goss arrested attention. A careful investigation was at once commenced. All the measurements of the body, muscular development, figure and general appearance corresponded accurately with the well-known description of Winfield S. Goss. The teeth were remarkably good, regular, even and well preserved. The remains were fully identified by Baltimore citizens who knew Goss intimately during his lifetime.

Udderzook was arrested July 15th, and on the 21st of October following the case came to trial.

The indictment contained two counts: one charging the prisoner with the murder of W. S. Goss, the other charging him with the murder of a person unknown. The defendant's consel insisted that the prosecution should decide upon which count it would proceed. The district attorney boldly announced his resolution to elect the count for the murder of W. S. Goss. The government thus assumed the double burden of proving not only the murder, but also the identity of the murdered man with one who there was at least strong evidence to show had died a year and a half before the alleged commission of the crime. The charge of killing W. S. Goss, however, opened at once an immense field of testimony otherwise incomplete and altogether meaningless in which the motive became abundantly apparent. Obviously by the fearless assumption of the greater burden lay also the greater, indeed the only, assurance of success.

The government made out an elaborate and perfect narrative, no material part of which was powerfully assailed by the prisoner. Udderzook really presented nothing worthy to be called a defence. He did not produce A. C. Wilson, (*alias* W. S. Goss) or any trace of him ; he did not explain who Wilson was, or what was the origin of his own acquaintance or the nature of his own connection with that mysterions person who was allowed across the scene, coming out of mystery, and in a few months plunging again into even more profound obscurity and during his brief sojourn in the known world proved to have had prior acquaintance with no persons save Udderzook and A. C. Goss.

Dr. E. W. Bailey testified : "I found the front teeth, the four upper incisors and the four below had been driven back into the mouth, two of them were lying loose on the tongue and the others were adhering. I removed them all from the mouth and have kept them in my possession. The person had what I would call a very good set of teeth ; they were firm and large and appeared healthy and strong.

Dr. E. Lloyd Howard. The upper front teeth had been driven back into the mouth, carrying with them a part of the sockets of the teeth. I found ten teeth remaining in the upper jaw and open, fresh sockets from which four others had been removed recently. Two upper teeth had been lost previous to death. In the lower jaw I found nine teeth remaining in position, and evidence that five others had been lost immediately after death or immediately preceding it. There were evidences of two lower jaw teeth having been lost some months previous to death. At the time of death he must have had twenty-eight teeth in all remaining in his mouth. The teeth lost previous to death, both in the upper and lowe jaw, were back teeth. The general appearance and character of h i teeth were perfectly good. They were white, even and regular. There were three or four gold fillings. and there were slight marks of disease pon two teeth."

The evidence completely overwhelmed Udderzook on his trial. The verdict was murder in the first degree.

The counsel for the defendant had taken a great many exceptions all relating to facts and circumstances bearing on the question of identity, but not one of them was ultimately sustained by the Supreme Court of Pennsylvania. Their multitude only denotes that the circumstances were numerous and in this multiplication consists the strength of the proof.

Chief-Justice Agnew, in delivering the opinion said: The great question in the case was the identity of A. C. Wilson as W. S. Goss. A variety of circumstances and many witnesses established beyond a doubt both the fact of this identity, and the fact that the body found in Bær's Woods was that of Goss.

And now in conclusion, if it should fall to our lot to speak as scientific experts, we can not do better than bear in mind the sensible advice of Sir William Blizzard. He says: "Be the plainest men in the world in a court of justice. Never harbor a thought that if you do not appear positive, you must appear little and mean. Give your opinion in as concise, plain and yet clear a manner as possible. Be intelligent, candid, and just, but never aim at appearing unnecessarily scientific. State all the sources by and from which you have gained your information. If you can, make your evidence a self-evident truth. This, though the court may at the time have too good or too mean an opinion of your judgment they must deem you an honest man. Never be dogmatic or set yourselves up for judge and jury. Take no side whatever, but be impartial and you will be honest."

NOTE.—This paper is introductory, rather than exhaustive. Other cases, are known to exist, notably that of the Prince Imperial (1879) but as full particulars could not be obtained in time for this publication they are not included. The writer respectfully asks his professional brethren and others to communicate to him any illustrative cases with which they are familiar, not here recorded. He proffers his grateful acknowledgment to Dr. C. C. Bombaugh for encouragement and for the use of his work: "Stratagems and Conspiracies to defraud Life Insurance Companies."

